# The association of DAT gene methylation with striatal DAT availability in healthy subjects

**DOI:** 10.1186/s13550-021-00800-y

**Published:** 2021-06-12

**Authors:** Kyoungjune Pak, Ju Won Seok, Hyun-Yeol Nam, Seongho Seo, Myung Jun Lee, Keunyoung Kim, In Joo Kim

**Affiliations:** 1 Department of Nuclear Medicine, Pusan National University Hospital and School of Medicine, Pusan National University, Busan, Republic of Korea; 2 Department of Nuclear Medicine, Chung-Ang University College of Medicine, Seoul, Republic of Korea; 3 Department of Nuclear Medicine, Samsung Changwon Hospital, Sungkyunkwan University School of Medicine, Changwon, Republic of Korea; 4 Department of Electronic Engineering, Pai Chai University, Daejeon, Republic of Korea; 5 Department of Neurology, Pusan National University Hospital, Busan, Republic of Korea

**Keywords:** Dopamine plasma membrane transport proteins, Methylation, Neuroimaging

## Abstract

**Background:**

DNA methylation inhibits gene expression by preventing transcription factors from binding to DNA. Functioning of nigrostriatal dopaminergic neurons is influenced by the expression of the dopamine transporter (DAT), and genetic variations in the gene encoding DAT contribute to differences in reward processing. We aimed to investigate the action of DAT methylation on DAT protein expression measured by positron emission tomography (PET).

**Methods:**

The emission data were acquired over 90 min with 50 frames after injection of ^18^F-FP-CIT using PET. Binding potentials (BP_ND_s) of ventral striatum, caudate nucleus, putamen were measured with the simplified reference tissue method. Genomic DNA was extracted from subjects’ blood sampling. Methylation of 4 regions in SLC6A3 gene was assessed using bisulfite pyrosequencing. The mean percentage of methylation (%) for each cluster was calculated by taking the average of all CpG site methylation levels measured within the cluster. Subjects were assessed with the Generalized Reward and Punishment Expectancy Scales (GRAPES) that consists of 30 items related with the reward and punishment that individuals expect for their behaviors.

**Results:**

Thirty-five healthy males, with an age range between 20 and 30 years, and a mean age of 24.4 ± 2.7 years, were included in this study. The mean percentage of methylation (%) from cluster C showed a trend of positive correlation with DAT availability of ventral striatum (rho = 0.3712, *p* = 0.0281), not significant after correction for multiple comparisons, and a significant correlation with GRAPES A: reward expectancy scale (rho = 0.7178, *p* < 0.0001).

**Conclusion:**

DAT methylation from peripheral blood showed a trend of positive correlation with DAT availability of ventral striatum in healthy subjects; however, it was not significant after correction for multiple comparison. The degrees of methylation from cluster C of DAT in peripheral blood were significantly correlated with reward scales of GRAPES A: reward expectancy scale. The association between DAT methylation and DAT expression needs to be investigated further.

## Background

The dopamine transporter (DAT) is a sodium chloride-dependent transmembrane protein on the presynaptic dopaminergic nerve terminal [1], regulating levels of dopamine by reuptake [2, 3]. As DAT binding is correlated with the density of dopaminergic neurons [4], decreased DAT uptake represents dopaminergic neurodegeneration such as PD [4], a neurodegenerative disease with death of dopaminergic neurons in the substantia nigra pars compacta [5, 6]. The most sensitive imaging techniques for the diagnosis of neurodegeneration are positron emission tomography (PET) and single photon emission computed tomography (SPECT) by using ligands that report nigrostriatal dopaminergic function [3], which allow quantifying dopaminergic system in human brain [7].

Epigenetic change is a heritable phenotype change without alterations in the DNA sequence, such as DNA methylation. DNA methylation is an epigenetic process that adds methyl groups to cytosines of the genome, mainly in CpG sites [8]. As DNA methylation inhibits gene expression by preventing transcription factors from binding to DNA [9], DNA methylation is inversely associated with protein expression [10]. Functioning of nigrostriatal dopaminergic neurons is influenced by DAT, and genetic variations in the gene encoding DAT contribute to differences in reward processing [11, 12]. However, in a previous study by Wiers et al., striatal DAT availability measured by ^11^C-cocaine PET did not show any significant association with DAT methylation of peripheral blood in healthy subjects [13], which is the only report that investigated the association between them. Therefore, we aimed to investigate the action of DAT methylation on DAT protein expression measured by PET and reward scales measured with the Generalized Reward and Punishment Expectancy Scales (GRAPES) [14].

## Methods

### PET scan

Thirty-five healthy, male subjects without brain injury, neuropsychological disorders were included in this study. The majority of the participants in this study were included in a previous study [15]. An intravenous bolus injection of ^18^F-FP-CIT was administered. The emission data were acquired over 90 min with 50 frames of progressively increasing durations (15 s × 8 frames, 30 s × 16 frames, 60 s × 10 frames, 240 s × 10 frames, and 300 s × 6 frames) using the Siemens Biograph 40 Truepoint PET/CT (Siemens Healthcare, Knoxville, Tennessee, USA). The dynamic PET data were collected in the 3-dimensional mode, with 148 slices with image sizes of 256 × 256 and pixel sizes of 1.3364 × 1.3364 mm^2^. These were reconstructed using iterative method with a Gaussian filter.

### Image analysis

For a volume-of-interest-based analysis, an averaged image (0–10 minafterinjection) was created from dynamic PET frames and spatially normalized to ^15^O-Water PET template in statistical parametric mapping 5 (Wellcome Trust Centre for Neuroimaging, United Kingdom). To extract time–activity curves (TACs) of volume of interest from full dynamic PET scans, Oxford-GSK-Imanova striatal atlas (https://fsl.fmrib.ox.ac.uk/fsl) for ventral striatum, caudate nucleus, putamen were applied. DAT availability, expressed in terms of binding potential (BP_ND_), was measured by analyzing TACs with the simplified reference tissue method [16] with the cerebellum as a reference using pmod version 3.6 (PMOD Technologies LLC, Zurich, Switzerland).

### DNA extraction and methylation analysis

Genomic DNA was extracted from subjects’ blood sampling. Methylation of 4 regions in SLC6A3 gene was assessed using bisulfite pyrosequencing. After bisulfite treatment of the DNA (EpiTect Fast DNA Bisulfite Kit, Qiagen, Hilden Germany), polymerase chain reaction test was done with DNA Engine Tetrad 2 Peltier Thermal Cycler (Bio-Rad, CA, USA). Primer Design was designed with PyroMark Assay Design 2.0 (Qiagen, Hilden Germany). Pyrosequencing was done with PyroMark Q48 Autoprep System (Qiagen, Hilden Germany). PyroMark Q48 Autoprep 2.4.2 Software (Qiagen, Hilden Germany) was used to assess methylation of each CpG site, 4 regions in SLC6A3 gene. The percentage of methylated alleles was divided by the total number of alleles (methylated and unmethylated) to determine the level of methylation at each CpG site. The mean percentage of methylation (%) for each cluster was calculated by taking the average of all CpG site methylation levels measured within the cluster.

### Questionnaire

Subjects were assessed with GRAPES [14]. GRAPES consists of 30 items related with the reward and punishment that individuals expect for their behaviors, GRAPES (A) reward expectancy, GRAPES (B) punishment expectancy.

### Statistical analysis

Normality was assessed using the D’Agostino & Pearson normality test. Spearman correlation test was done to determine the association of DAT methylation with BP_ND_s and GRAPES. As 4 clusters of DAT methylation were included in this study, significance was set at *ɑ* = 0.05/4 (0.0125) to correct for multiple comparisons. All analyses were conducted using Prism (v7.0d, GraphPad Software Inc, La Jolla, CA, USA).

## Results

**Fig. 1:**
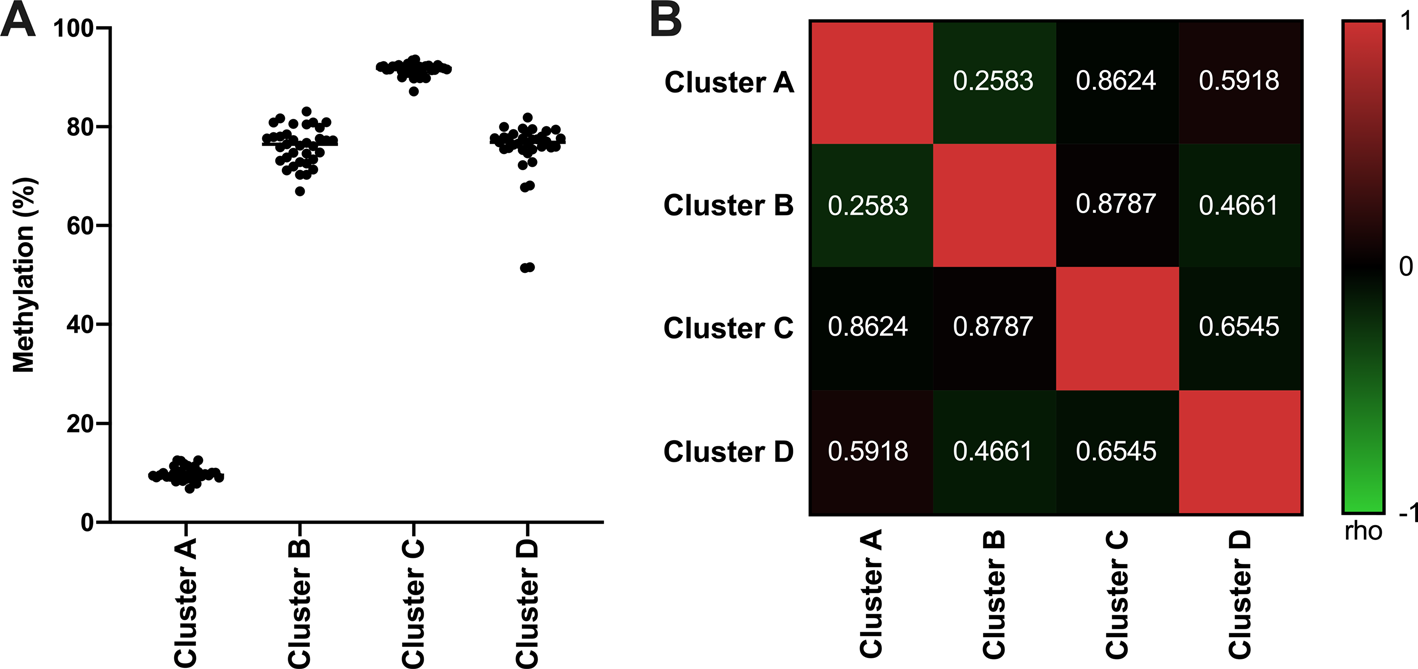
**a** Distribution of mean percentage of methylation (%) for each cluster and **b** inter-correlation of mean percentage of methylation (%)

**Fig. 2:**
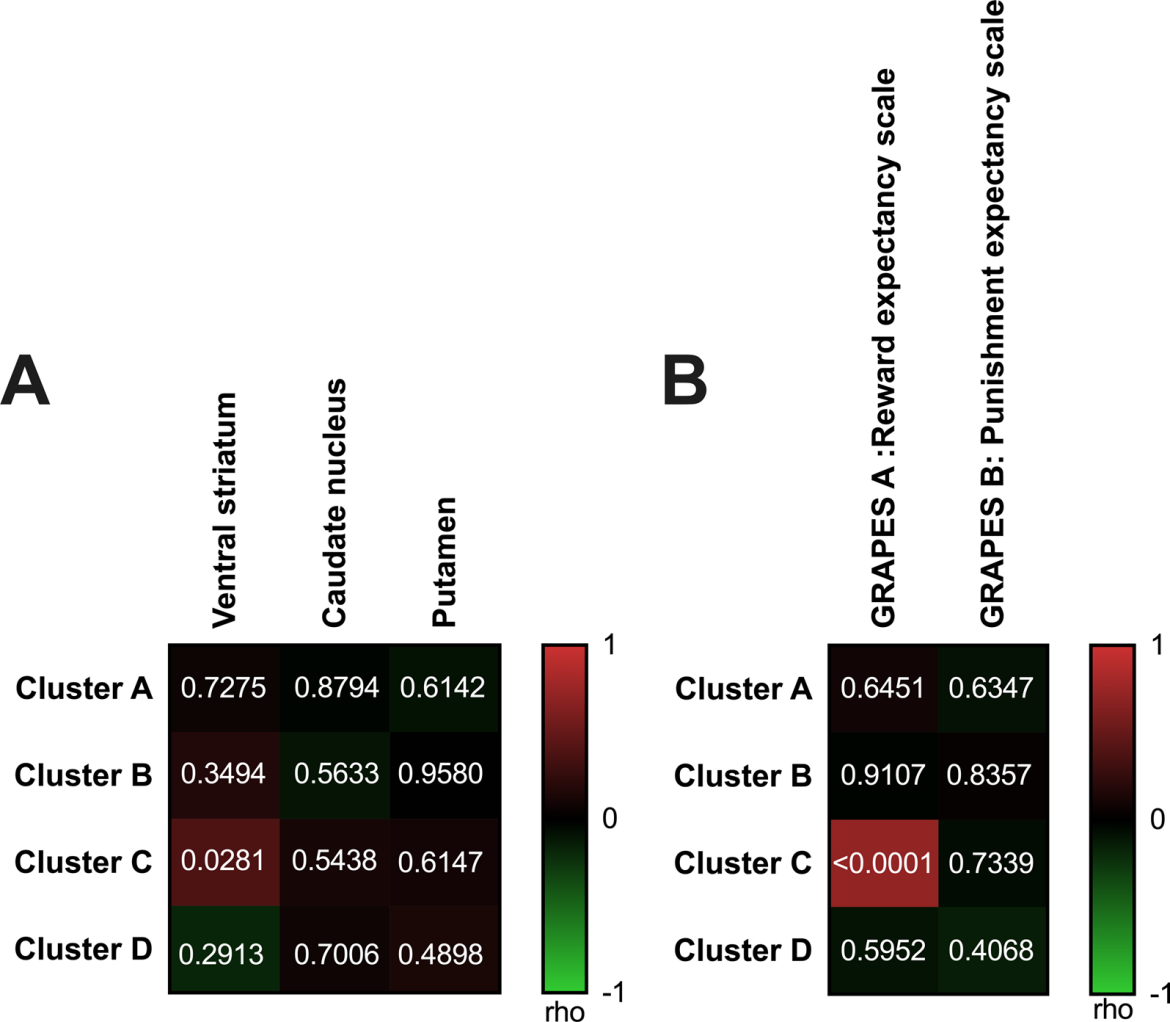
Correlation of mean percentage of methylation (%) with **a** DAT availability and **b** GRAPES

**Fig. 3:**
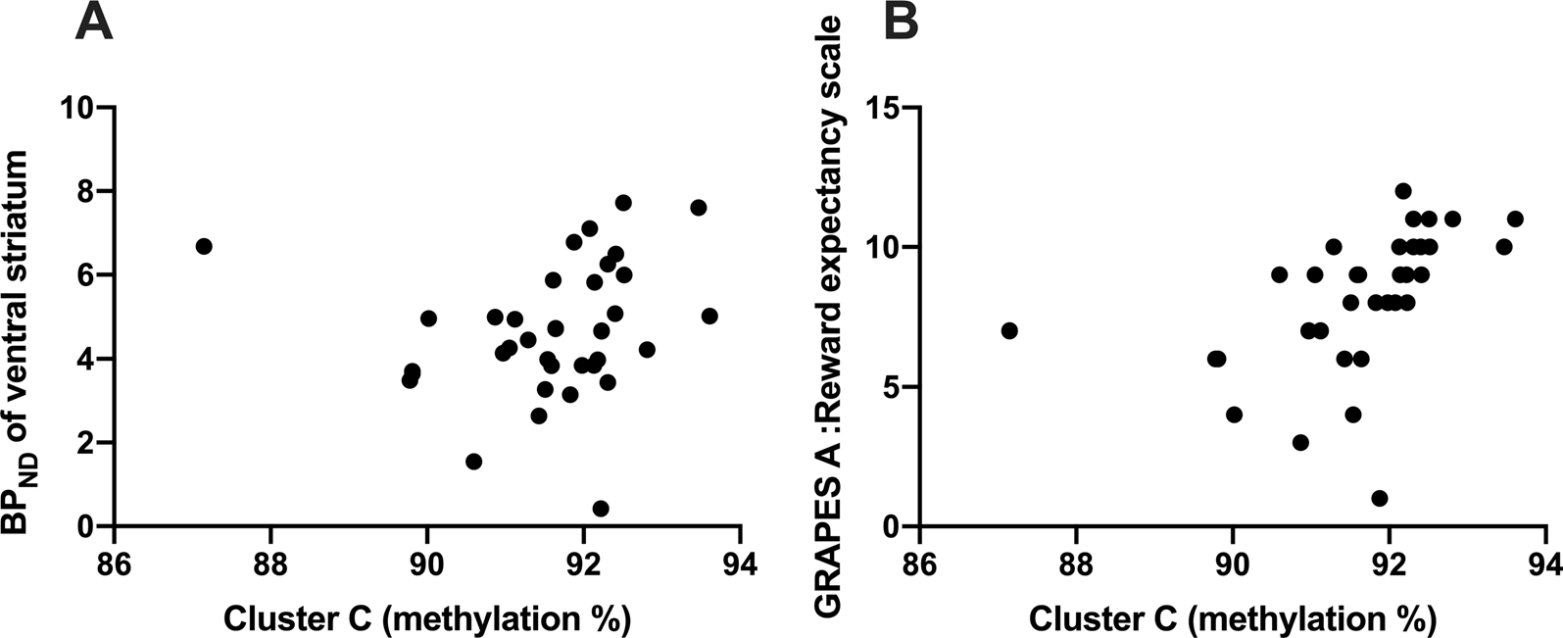
Correlation of mean percentage of methylation (%) from cluster C with **a** DAT availability and **b** GRAPES

Thirty-five healthy males, with an age range between 20 and 30 years, and a mean age of 24.4 ± 2.7 years, were included in this study. Coordinates and the percentage of methylated alleles (%) for all SLC6A3 CpG sites are presented in Table 1. The mean percentages of methylation (%) were 9.9 (cluster A), 76.1 (cluster B), 91.6 (cluster C), 75.1% (cluster D), respectively. The mean percentage of methylation (%) from each cluster did not show any association each other (Fig. 1). The mean percentage of methylation (%) from each cluster was not associated with DAT availability of the ventral striatum, showing a trend of positive correlation between that from cluster C and DAT availability of the ventral striatum without correction (rho = 0.3712, *p* = 0.0281), which was not significant after correction for multiple comparison. In addition, the mean percentage of methylation (%) from cluster C was positively correlated with GRAPES A: reward expectancy scale (rho = 0.7178, *p* < 0.0001) (Figs. 2, 3).

**Table 1:** Coordinates and the percentage of methylated alleles (%) for all SLC6A3 CpG sites

Assay ID	CpG ID	Cluster	From ATG	From TSS	GRCh37/hg19	Region	The percentage of methylated alleles (%)
ADS2165RS2	237	A	− 3177	− 944	1446489	Promoter	13.43
ADS2165RS2	236	A	− 3174	− 941	1446486	Promoter	16.98
ADS2165RS2	235	A	− 3167	− 934	1446479	Promoter	9.30
ADS2165RS2	234	A	− 3163	− 930	1446475	Promoter	10.15
ADS2165RS2	233	A	− 3151	− 918	1446463	Promoter	6.03
ADS2165RS2	232	A	− 3148	− 915	1446460	Promoter	9.79
ADS2165RS1	231	A	− 3134	− 901	1446446	Promoter	8.85
ADS2165RS1	230	A	− 3132	− 899	1446444	Promoter	4.38
ADS2127FS	547	B	26,817	29,050	1416496	Intron 6	79.69
ADS2127FS	548	B	26,856	29,089	1416504	Intron 6	72.46
ADS2126FS2	551	C	27,008	29,241	1416305	Exon 7, Intron 6	95.82
ADS2126FS2	552	C	27,010	29,243	1416303	Exon 7, Intron 6	88.41
ADS2126FS	553	C	27,038	29,271	1416275	Exon 7, Intron 6	98.75
ADS2126FS	554	C	27,047	29,280	1416266	Exon 7, Intron 6	83.28
ADS2796FS1	1149	D	48,635	50,868	1394678	Exon 15 3’UTR	70.80
ADS2796FS1	1150	D	48,640	50,873	1394673	Exon 15 3’UTR	96.21
ADS2796FS1	1151	D	48,646	50,879	1394667	Exon 15 3’UTR	65.53
ADS2796FS1	1152	D	48,653	50,886	1394660	Exon 15 3’UTR	78.86
ADS2796FS1	1153	D	48,656	50,889	1394657	Exon 15 3’UTR	62.88
ADS2796FS2	1154	D	48,679	50,912	1394634	Exon 15 3’UTR	69.95
ADS2796FS2	1155	D	48,682	50,915	1394631	Exon 15 3’UTR	69.51
ADS2796FS2	1156	D	48,697	50,930	1394616	Exon 15 3’UTR	92.09
ADS2796FS2	1157	D	48,709	50,942	1394604	Exon 15 3’UTR	70.38

## Discussion

In this study, the degrees of methylation from cluster C (Exon 7) of DAT in peripheral blood showed a trend of positive correlation with DAT availability of the ventral striatum and were significantly correlated with reward scales of GRAPES A: reward expectancy scale.

This is the first study that investigated the association between the degree of methylation of DAT and DAT availability of PET in healthy subjects. Previously, the association of methylation of DAT gene with DAT availability was investigated in patients with attention-deficit hyperactivity disorder (ADHD) [13], which is the only study of DAT methylation and PET. Although methylation of DAT gene was not significantly different between patients with ADHD and healthy subjects, DAT availability measured by ^11^C-cocaine PET was negatively correlated with the degree of DAT methylation in patients with ADHD, not in healthy subjects [13]. This correlation was significant in both caudate nucleus, putamen, and ventral striatum [13]. However, the underlying mechanisms of insignificancy between DAT availability and DAT methylation in healthy subjects remained unclear. In subjects with alcohol dependency, the effect of DAT methylation was investigated with reward processing [10] and depressive symptoms [17] using functional magnetic resonance imaging. DAT methylation in promoter region predicted activation of nucleus accumbens during the anticipation of high and low loss in healthy subjects, not in subjects with alcohol dependency [10]. In addition, subjects with alcohol dependency showed that activity of amygdala to alcohol cue was correlated with DAT methylation only those with low depression scores [17]. Also, higher DAT methylation predicted the relapse of alcohol dependency [17].

Epigenetic change is a heritable phenotype change without alterations in the DNA sequence, such as DNA methylation. DNA methylation is a process that adds methyl groups to cytosines of the genome, mostly in CpG sites [8]. DNA methylation inhibits gene expression by preventing transcription factors from binding to DNA [9]. Therefore, DNA methylation has shown to be inversely associated with protein expression [10]. In rodent, DAT methylation was negatively associated with striatal DAT expression [18]. In humans, only one study reported the association between DAT methylation and DAT availability measured by ^11^C-cocaine PET as explained above [13]. Inconsistent with the study by Wiers et al., we demonstrated a trend of positive association between DAT methylation and DAT availability measured by ^18^F-FP-CIT PET. In addition, the mean percentage of methylation (%) from cluster C was strongly correlated with GRAPES A: reward expectancy scale, similar to the study by Muench et al., which predicted striatal responses during reward processing by DAT methylation in healthy subjects, not in alcohol-dependent individuals [10]. DAT methylation in alcohol-dependent individuals [10], or ADHD [13], showed the difference with that in healthy subjects. Therefore, DAT methylation might have an implication on treatment of alcohol dependency or ADHD.

There are several points that should be considered in these results. First, it is unclear that whether DAT methylation obtained from peripheral blood has an association with DAT methylation of brain. In this regard, Wiers et al. proved the significant correlation between DAT methylation of substantia nigra and DAT methylation from peripheral blood both in healthy subjects and patients with ADHD, however, showing moderate correlation in both promotor region (*r* = 0.44), and CpG site 230 (*r* = 0.53) of healthy subjects [13]. Second, the mean percentage of methylation was calculated from each cluster and considered as the degree of DAT methylation level. However, the degree of DAT methylation may be different within each cluster, and the region of each cluster may be different. Third, the radiopharmaceuticals used in measurement of DAT availability were different in each study. Theoretically, as DNA methylation inhibits or regresses the binding of transcription factors [9], DNA methylation might be negatively associated with protein expression [10]. However, at least with regard to DAT, this association is unclear until now as we showed a trend of positive association between DAT methylation and DAT availability, and Wiers et al. showed no significant association [13]. The mechanisms underlying these findings might be complex and likely vary across genes. Also, as a small number of subjects were included in this study, further studies of a larger number of subjects with a broader age range should be carried out in the future.

## Conclusion

DAT methylation from peripheral blood showed a trend of positive correlation with DAT availability of ventral striatum in healthy subjects; however, it was not significant after correction for multiple comparison. The degrees of methylation from cluster C of DAT in peripheral blood were significantly correlated with reward scales of GRAPES A: reward expectancy scale. The association between DAT methylation and DAT expression needs to be investigated further.

## Data Availability

All data are available to corresponding author of the manuscript upon reasonable request.
