# Correction: The association of DAT gene methylation with striatal DAT availability in healthy subjects

**DOI:** 10.1186/s13550-023-00991-6

**Published:** 2023-05-10

**Authors:** Kyoungjune Pak, Ju Won Seok, Hyun-Yeol Nam, Seongho Seo, Myung Jun Lee, Keunyoung Kim, In Joo Kim

**Affiliations:** 1 Department of Nuclear Medicine, Pusan National University Hospital and School of Medicine, Pusan National University, Busan, Republic of Korea; 2 Department of Nuclear Medicine, Chung-Ang University College of Medicine, Seoul, Republic of Korea; 3 Department of Nuclear Medicine, Samsung Changwon Hospital, Sungkyunkwan University School of Medicine, Changwon, Republic of Korea; 4 Department of Electronic Engineering, Pai Chai University, Daejeon, Republic of Korea; 5 Department of Neurology, Pusan National University Hospital, Busan, Republic of Korea

**Correction to: EJNMMI Research (2021) 11:58** 10.1186/s13550-021-00800-y

Following publication of the original article [1], it came to the corresponding author's attention that affiliation 1 had been incorrectly detailed in the article. The published article has been updated to correct the affiliation and the corrected affiliation may be seen in this erratum. The authors thank you for reading and apologize for any inconvenience caused.
